# Effect of the autoimmune-associated genetic variant *PTPN22* R620W on neutrophil activation and function in patients with insulin-dependent diabetes mellitus

**DOI:** 10.3389/fimmu.2025.1554570

**Published:** 2025-09-08

**Authors:** Eugenia Belcastro, Annamaria Cudini, Irene Mezzani, Stefania Petrini, Valentina D’Oria, Riccardo Schiaffini, Marco Scarsella, Anna Lo Russo, Alessandra Fierabracci

**Affiliations:** ^1^ Bambino Gesù Children’s Hospital, Istituto di Ricovero e Cura a Carattere Scientifico (IRCCS), Rome, Italy; ^2^ Confocal Microscopy Core Facility, Bambino Gesù Children’s Hospital, Istituto di Ricovero e Cura a Carattere Scientifico (IRCCS), Rome, Italy

**Keywords:** type 1 diabetes, variant PTPN22, reactive oxygen species, neutrophils activation, endothelial cells

## Abstract

**Introduction:**

Recent evidence highlights neutrophils’ role in initiating/sustaining aberrant immune responses in type 1 diabetes (T1D). The *PTPN22* C1858T variant, a risk factor for several autoimmune conditions including T1D, affects T/B-cell receptor signaling. This study investigates its contribution to the altered neutrophil activation and function in T1D.

**Materials and methods:**

Neutrophils were isolated from peripheral blood mononuclear cells (PBMCs) of wild-type (WT) C1858C, heterozygous (HET) C1858T T1D patients, and healthy controls (HD). Reactive oxygen species (ROS) levels were assessed using a fluorogenic probe by Fluorescence- Activated Cell Sorting (FACS) in tumor necrosis factor-alpha (TNF-α) primed, unprimed-*N*-formylmethionyl-leucyl-phenylalanine (fMLP) stimulated, and primed-stimulated neutrophils. Neutrophil adhesion/transmigration was evaluated via brightfield and epifluorescence microscopy on human umbilical vein endothelial cells (HUVECs).

**Results:**

Neutrophil counts were increased in the female patients than in HD. ROS production was enhanced in the neutrophils of the HET patients *versus* WT controls under the different culture conditions. Furthermore, ROS levels were increased in the HET (n = 10) *versus* WT (n = 6) patients (p < 0.05). Neutrophil adhesion to HUVECs and transmigration through the monolayer were increased in the HET (n = 4) *versus* WT (n = 6) patients under both basal and TNF-α conditions (p < 0.0001).

**Conclusion:**

Neutrophils from C1858T patients are more intrinsically active with increased ROS production and HUVEC adhesion/transmigration, suggesting enhanced contribution to the migration of other immunotypes from vessels into the pancreatic islets during T1D etiopathogenesis.

## 1 Introduction

Type 1 diabetes (T1D) is recognized as a multifactorial etiopathogenesis with several gene polymorphisms affecting the regulation of the innate and adaptive immune responses (1, 2). Half of the genetic risk is linked to the Human Leukocyte Antigen (HLA) region, i.e., class II and class I genes (3). Outside the HLA region, the insulin variable number of tandem repeats (*INS*-VNTR) polymorphism in the *INS* promoter displays a stronger T1D association (4). Other robustly linked genes are cytotoxic T lymphocyte-associated antigen 4 (*CTLA4*) (5), protein tyrosine phosphatase N22 (*PTPN22*) (6), and interleukin-2 receptor subunit alpha (*IL2RA*)https://www.genecards.org/cgi-bin/carddisp.pl?gene=IL2RA (7). In recent years, the idea that autoimmune diseases are mediated exclusively by autoreactive T lymphocytes, the predominant immunotype observed at histopathology of a few reported cases of early-stage human insulitis (8), has been revised (9). Macrophages and neutrophils, which physiologically reside in the pancreatic islet, participate in tissue homeostasis and organ damage, playing a central role in the initiation and perpetuation of aberrant immune responses (10). During the development of insulitis, a strong interaction occurs between cells of innate and adaptive immunity (10). Proteins released from beta (β) cells are taken up and processed by innate immune cells and presented to autoreactive T lymphocytes against the pancreatic islet after their migration into the draining lymph nodes (11). β-Cell death, in turn, triggers the recruitment and activation of B1a cells, neutrophils, and plasmacytoid dendritic cells (12).

In T1D, reduced circulating neutrophil numbers precede and accompany the disease, a phenomenon that parallels the active autoimmune destruction of β-cells (13). Furthermore, β-cell function declines more significantly in individuals with the lowest percentages of circulating neutrophils. Neutrophils infiltrate the pancreas and extrude neutrophil extracellular traps in coordination with other immune cells, even prior to the onset of clinical symptoms in presymptomatic at-risk subjects (13). Neutrophils direct and guide the innate immune response through complex interactions with macrophages, natural killer cells (14), dendritic cells, and effector cell mediators, i.e., soluble recognition molecules, which have the property of increasing phagocytosis, stimulating the complement system, and modulating inflammation, cytokine production, tissue damage molecules, and pathogens that contribute to the innate inflammatory environment (15, 16).

The reduction of circulating neutrophils in T1D subjects can be caused by their impaired reduced bone marrow egression compared with that in non-diabetic subjects (17), a feature that in mice was associated with a defective bone marrow microenvironment (17). Additionally, the heightened consumption and destruction of circulating neutrophils can be attributed to increased apoptosis or anti-neutrophil specific antibodies and increased homing and recruitment to the target organ (18, 19). The reduced number of circulating neutrophils can also be related to the possible triggering of viral infections that initiate and accelerate the process of islet autoimmunity (20).

Among the genetic polymorphisms that regulate the innate and adaptive immune responses in T1D etiopathogenesis, the C1858T variant of *PTPN22* is a prominent risk factor for several autoimmune conditions, altering signaling through T- and B-cell receptors (21–23), thus supporting the survival of autoreactive T lymphocytes. In particular, in humans, this variant is significantly associated with T1D, augmenting the risk of developing the disorder by two- to fourfold (24). The Lyp protein encoded by *PTPN22* controls the effect of tyrosine kinases and regulates signaling pathways by phosphorylating tyrosine residues in target proteins (22). The Lyp protein has an effect not only on lymphocytes but also on neutrophils, where it is highly expressed (25). The variant is responsible for a more precocious onset of T1D compared to WT patients and more severe clinical manifestations due to a rapid decline in the β-cell reservoir. The variant halts the pathogenetic mechanism, causing a “gain of function” in the encoded Lyp protein with a paradoxical reduced T-cell activation (21).

This study aimed to investigate the contribution of the C1858T *PTPN22* variant encoding the Lyp variant protein (R620W) in the altered activation and function of neutrophils in T1D. Indeed, altered tyrosine phosphorylation levels were observed in neutrophils (26), and enhanced neutrophil activation and function have been reported in patients affected by rheumatoid arthritis harboring variant *PTPN22* (27). In the present investigation, the impact of the variant on reactive oxygen species (ROS) production by neutrophils (28) and their adhesion and transmigration properties to endothelial cells was specifically evaluated.

## 2 Materials and methods

### 2.1 Subjects

Neutrophil counts were assessed in 74 T1D patients recruited at onset and 66 healthy blood donors (**Supplementary Tables 1**, **2**). Among the T1D patients, 37 harbored the C1858T *PTPN22* variant, while 37 were wild-type (WT). For in-depth experimental analyses of neutrophil activation and function, a cohort of 27 unrelated T1D patients (13 female and 14 male patients with an age range at referral of between 1.1 and 16.8 years) were recruited from the University Department of Pediatrics (DPUO) at Bambino Gesù Children’s Hospital (OPBG) in Rome (**Table 1**). Fifteen healthy blood donors representing the control group were recruited from the OPBG Blood Transfusion Centre (**Supplementary Table 2**). The overall experimental design is illustrated in **Supplementary Figure 1**.

**Table 1:** Clinical characteristics of T1D patients (n = 27) included for in-depth experimental analyses.

No.	Gender	Actual age (years)	Age at onset	Clinical manifestation	Auto Abs	*PTPN22* genotype	C-peptide at onset (ng/mL)	HbA1c at onset (mmol/mol)
1	F	12.9	10.4	T1D	**GADA**, **IA2Ab**, **ZnT8pos** IAA, TRGAb, TPOAb neg	HET	0.67	111
2	F	15.6	11	T1D	**GADA, IA2Ab, ZnT8 pos** IAA, TRGAb, EMA, TgAb, TPOAb, TgAb, TPOAb, TRAb, DGP-IgGAb neg	WT	2.8	52
3	M	18.3	11.5	T1D	**GADA, IA2Ab pos** IAA, ZnT8, TRAb, TgAb, TPOAb, TRGAb, DGP-IgGAb, ANA, anti-DNA, ENA, Rab neg	WT	0.23	117
4	M	9.7	7	T1D	**GADA pos** IA2Ab, IAA, ZnT8, TgAb, TPOAb, TRGAb, EMA neg	WT	0.6	79
5	F	13.3	6.5	T1D	**IA2Ab, TPOAb pos** GADA, IAA, TgAb, TRGAb, EMA neg	WT	0.40	120
6	M	17.5	16.8	T1D	**GADA pos,** IA2Ab, IAA, ZnTIAA, ZnT8, TgAb, TPOAb, TRGAb, EMA neg	WT	1.43	98
7	M	15.7	7.1	T1D	GADA, IA2Ab, IAA, ZnT8, TgAb8, TgAb, TPOAb, TRGAb, TPOAb, TRGAb, EMA neg	HET	NA	NA
8	F	12.6	2.2	T1D high cholesterol levels (familial hypercholesterolemia)	**GADA, IAA, TRGAb-IgA pos** IA2Ab, TgAb, TPOAb, DGP-IgGAb, EMA neg	HET	0.08	66
9	M	16.6	6	T1D	**IAA pos** GADA, IA2Ab, TgAb, TPOAb, TRGAb, DGP-IgGAb, EMA neg	HET	NA	58
10	M	17.8	2	T1D	**IAA pos** GADA, IA2Ab, TgAb, TPOAb, TRGAb, DGP-IgGAb, EMA neg	WT	NA	NA
11	F	11.1	6.4	T1D	**ZnT8 pos** GADA, IA2Ab, IAA, TgAb, TPOAb, TRGAb, DGP-IgGAb, EMA neg	HET	0.47	108
12	F	17.5	12.7	T1D, autoimmune thyroiditis	**GADA**, **IA2Ab**, **TgAb**, **TPOAb pos** IAA, TRGAb, DGP-IgGAb neg	HET	0.46	117
13	M	12	1.1	T1D	**GADA, IA2Ab, IAA pos** TgAb, TPOAb, TRGAb neg	HET	0.35	62
14	F	18.6	7.1	T1D	**GADA, IA2Ab pos** IAA, TgAb, TPOAb, TGRAb, DGP-IgGAb neg	HET	0.23	59
15	M	18.5	10.1	T1D	**GADA, IA2Ab, IAA pos** TgAb, TPOAb, TRGAb, DGP-IgGAb neg	HET	0.39	119
16	M	14.6	4.8	T1D	**GADA, IA2Ab, IAA pos,** TPOAb **pos**, TPOAb, TgAb, TRGAb, DGP-IgGAb, ANA, ANCA, ASCA neg	HET	0.2	106
17	M	14.5	7.1	T1D	**GADA, IA2Ab, IAA pos** TgAb, TPOAb, TRGAb, DGP-IgGAb neg	HET	0.46	86
18	F	18.6	2	T1D, hypovitaminosis	**GADA,IAA, TRGAb**, **EMA pos** IA2Ab, TgAb, TPOAb, DGP-IgGAb neg IgGAb neg	HET	0.06	96
19	M	21	11	T1D	**GADA, IA2Ab, IAA pos** TgAb, TPOAb, TRAb, TRGAb, DGP-IgGAb neg	HET	0.41	108
20	F	14.5	1.1	T1D, Basedow’s disease	**TgAb, TPOAb**, **TRAb pos** TRGAb neg	HET	NA	NA
21	F	12.4	1.9	T1D, celiac disease	**IA2Ab, IAA, TRGAb EMA-IgA pos** GADA, TgAb, TPOAb, DGP-IgGAb neg	HET	0.12	82
22	F	13.7	3.1	T1D	**GADA, IA2Ab, CAb, β2GP-1Ab, anti-phospholipid pos** IAA, TgAb, TPOAb, TRGAb, DGP-IgGAb, EMA-IgA, LKMAb, LC1Ab, ANA, anti-DNA, ENA, RAb, ARA, AMA, ASMA, APCA, ASMA, APCA, anti-adrenal neg	WT	0.27	NA
23	M	12.8	9.8	T1D, choroidal nevus	**IA2Ab, ZnT8,TGRAb pos** GADA, IAA, TgAb, TPOAb, DGP-IgGAb neg	WT	0.43	93
24	F	12.9	2	T1D, celiac disease	**GADA**, **IA2Ab, IAA,ZnT8, TRGAb-IgA**, **EMA-IgApos** TgAb, TPOAb, TRGAb neg	WT	NA	95
25	F	15	8.7	T1D	**GADA, IA2Ab, IAA pos** TgAb, TPOAb, TRGAb, TPOAb, TRGAb, EMA neg	WT	0.51	84
26	M	15	10.4	T1D	**GADA, IA2Ab pos** IAA, ZnT8, TgAb, TPOAb, TRGAb, DGP-IgGAb neg	WT	0.25	128
27	M	9.3	5.4	T1D	**GADA pos** IA2Ab, IAA, TPOAb, IAA, TPOAb, TRGAb neg	WT	NA	NA

NA, not available; M, male; F, female; T1D, type 1 diabetes; HET, heterozygous; WT, wild type; HbA1c, Glycated Hemoglobin A1c.

Positive auto-antibodies are highlightdes in bold.

The patients’ sera were assayed for insulin-dependent diabetes mellitus (T1D)-related Abs, i.e., anti-glutamic acid decarboxylase isoform 65 (GADAb) (First anti-GAD ELISA RSR, Cardiff, UK), anti-tyrosine phosphatase-related islet antigen 2 (IA2Ab) (First antiIA2 ELISA RSR), anti-insulin (IAA) (Medzyme Corporation, Montreal, QC, Canada), and anti-zinc transporter 8 Abs (ZnT8Ab) (anti-ZnT8 RSR) by enzyme-linked immunosorbent assay (ELISA); and for thyroid-related Abs, i.e., anti-thyrotropin (TSH) receptor (TRAb) immunoassay (Immulite TSI, Siemens Healthcare, Tarrytown, NY, USA), thyroglobulin (TgAb), and thyroperoxidase (TPOAb) *via* electrochemiluminescence immunoassay (ECLIA) (Siemens, Erlangen, Germany). Celiac-disease-related Abs were screened by chemiluminescence (CLIA; Quanta-Flash-Werfen, Monza, Milan, Italy), i.e., anti-transglutaminase (TRGAb) (CLIA; Quanta-Flash-Werfen, Monza, Milan, Italy), and by fluorimetric enzyme-linked immunoassay (FEIA), endomysial Ab (EMA) (Werfen, Barcelona, Spain) by immunofluorescence (IFL), and anti-deamidated gliadin (DPG-IgG Ab) by EliA. Specific hepatic Abs, i.e., anti-liver–kidney microsomal (LKMAb) and anti-liver cytosol type 1 (LC1Ab), anti-ribosomal (RAb), anti-adrenal (ADR Ab), and parietal cell antibodies (APCA), were measured by indirect IFL (KSL-Werfen). Non-organ-specific Abs anti-nuclear (ANA), anti-neutrophil cytoplasmic (ANCA) (Elettrochimica s.r.l., Malnate, Varese), anti-double-stranded DNA (anti-dsDNA) (bioMérieux Italia S.p.A, Bagno a Ripoli, Florence, Italy), anti-mitochondrial (AMA), anti-smooth muscle (ASMA), and anti-reticulin Ab (ARA) were tested by IFL (KSL-Werfen). Extractable nuclear antigen Abs (ENA, SSA/RoAb) were tested by ELiA (Thermo Fisher, Waltham, MA, USA). Anti-cardiolipin (ACA) and anti-beta 2 glycoprotein 1 Ab (anti-β2GP1) were measured by ELISA. Informed consent was obtained from all those who took part in the present study in accordance with the Declaration of Helsinki. All subjects entered the study after written informed consent was obtained. The investigation was approved by the local institutional review board (IRB) of Bambino Gesù Children’s Hospital, which regulates human sample usage for experimental studies (Study protocol no. 2436_OPBG_2020); all procedures followed were in accordance with institutional guidelines. Written informed consent was obtained from the next of kin in the case of children. Participant consent was recorded using a paper-based inventory system. The IRB approved the consent procedure.

### 2.2 Screening for the presence of C1858T *PTPN22* variant

Peripheral blood from patients and healthy control donors was collected in Ethyleneidiaminetetraacetic acid (EDTA), and leukocyte genomic DNA was extracted from whole blood samples using QIAmp DNA blood mini kit (Qiagen, Hilden, Germany), according to the manufacturer’s guidelines. The detection of the C1858T variant in the *PTPN22* gene (protein tyrosine phosphatase N22, GenBank ID: 26191) was performed via PCR using specific primers for the amplification of exon 14 of the *PTPN22* gene: forward 5′-GATAATGTTGCTTCAACGGAATTT-3′ and reverse 5′-CCTCAAACTCAAGGCTCACAC-3′ (annealing temperature 58.5°C). The amplification lasted 35 cycles, generating PCR products of 318 bp that were purified using the NucleoSpin Gel and PCR Clean-up kit (Bioanalysis). Purified PCR product sequencing was carried out using the BigDye Terminator v.3.1 Cycle sequencing protocol (Life Technologies, Applied Biosystems, Paisley, Scotland, UK).

### 2.3 Neutrophil count

Neutrophil counts were analyzed in a cohort of 74 T1D patients and 66 age- and sex-matched healthy blood donors. Comparison was also conducted between 35 T1D female patients and 25 controls, and between 39 male T1D patients and 41 healthy donors. The mean age of healthy donors was less than 24 years. The mean age at referral for all T1D patients was 17.74 years, with a mean age at T1D onset of 8.03 years. Specifically, for the WT *PTPN22* T1D patients, the mean age at referral was 16.88 years, and the age at T1D onset was 7.88 years. For the heterozygous (HET) C1858T *PTPN22* patients, the mean age at referral was 18.49 years, with a mean onset age of 7.82 years. Neutrophil counts were also compared between 37 wild-type and 36 heterozygous *PTPN22* T1D patients *vs*. the healthy control group. Additionally, neutrophil counts were examined in a subset of diabetes-related autoantibody-positive WT T1D patients (n = 37) and HET for the *PTPN22* polymorphism (n = 37) *vs*. autoantibody-negative healthy controls (n = 66).

### 2.4 Isolation of peripheral blood neutrophils

Polymorphonuclear (PMN) leukocytes were isolated from whole blood samples collected in EDTA obtained from 27 patients and 15 controls using discontinuous gradient centrifugation according to English et al. (29). Briefly, each whole blood sample (approximately 6 mL) was carefully stratified over a double Pancoll human 1077 (PAN-Biotech GmbH, Aidenbach, Germany)/Histopaque 1119 (Sigma-Aldrich Co., St Louis, MO, USA) gradient and centrifuged at 3,500 rpm for 40 minutes, with low acceleration and without brake at room temperature. Granulocyte ring was recovered; cells were washed twice with sterile 0.9% NaCl solution. Contaminated red blood cells were lysed by adding 2 mL of cold sterile H_2_O and gently mixing the tubes by inverting them for 20 seconds at room temperature. The cells were then immediately washed with 2% NaCl solution and resuspended in fresh phosphate-buffered saline (PBS) before being counted in a Burker chamber.

### 2.5 Reactive oxygen species assessment

The intracellular ROS levels were assessed using a 5-(and 6-)-chloromethyl-2′,7′-dichilorodihydrofluorescein diacetate, acetyl ester (CM-H_2_DCFDA; Life Technologies, Carlsbad, CA, USA) fluorogenic probe. Specifically, among the T1D cohort (**Table 1**), ROS analysis was performed in neutrophils isolated from 16 T1D patients. The mean age at referral for all T1D patients was 12.4 years, with a mean age at T1D onset of 5.9 years. Specifically, for the WT T1D patients, the mean age at referral was 11 years, and the age at T1D onset was 6.6 years. For the HET C1858T patients, the mean age at referral was 13.2 years, with a mean onset age of 5.5 years. For the healthy donor group, a subgroup of five individuals were included in the ROS production analysis. Neutrophils (1.0 × 10^6^/mL in PBS) were primed with 5 ng/mL tumor necrosis factor-alpha (TNF-α) (PeproTech, Cranbury, NJ, USA) for 15 minutes at 37°C and then incubated with 2 μM CM-H_2_DCFDA for 20 minutes at 37°C in the dark. Samples were also stimulated with 1 μM *N*-formylmethionyl-leucyl-phenylalanine (fMLP) (Sigma-Aldrich Co., USA) for 30 minutes at 37°C, followed by washing twice in PBS before flow cytometry analysis. Negative and positive controls were also included. Data were acquired using the BD LSRFortessa X-20 flow cytometer (BD Biosciences, Sean Jose, CA, USA) and revealed by mean fluorescence intensity (MFI) values. Dead cells were excluded from analysis by side/forward scatter (SSC/FSC) gating prior to propidium iodide staining. To evaluate potential age-dependent effects on neutrophil function, a correlation analysis was conducted between age and ROS production across all samples, as well as within the T1D patient subgroup.

### 2.6 Adhesion and transmigration assays

Neutrophil adhesion and transmigration through human umbilical vein endothelial cell (HUVEC; Life Technologies) monolayers was conducted in 10 T1D patients, with (n = 4) or without (n = 6) the C1858T *PTPN22* gene variant. The mean age at referral for all T1D patients was 14.4 years, with a mean age at T1D onset of 8.5 years. Specifically, for the WT T1D patients, the mean age at referral was 12.7 years, and the age at T1D onset was 9.1 years. For the heterozygous C1858T patients, the mean age at referral was 14.4 years, with a mean onset age of 6.4 years; within both groups (WT and HET), patients were age matched. HUVECs were cultured at recommended seeding density (2.5 × 10^5^ cells/cm^2^) in Endothelial Growth Medium-2 (EGM™-2, Euroclone, Milan, Italy), according to the manufacturing datasheet. For adhesion and transmigration assays, HUVECs (1.5 × 10^5^ cells in 300 μL) were seeded onto 24-well cellQART^®^ Cell Culture Inserts (3-μm PET Translucent, SABEU GmbH & Co. KG, Northeim, Germany) and cultured overnight to generate confluent monolayers. As previously described (30, 31), TNF-α (5 ng/mL) was added to confluent monolayers for 4 hours before the adhesion. The concentration of 5 ng/mL was chosen since it was shown as the most effective with neutrophils from C1858T patients with rheumatoid arthritis (27). Isolated neutrophils were then added for 2 hours at 37°C to allow adhesion and migration. The number of neutrophils added ranged from 1 × 10^5^ to 2 × 10^6^/mL, depending on the blood volume collected from each patient. After the removal of non-adherent neutrophils and washing the inserts with PBS, samples were fixed with 4% paraformaldehyde for 15 minutes in the dark. After two additional PBS washes, the cells’ nuclei were counterstained with Hoechst (1:5,000 in PBS) for 15 minutes. Digitized images of the endothelial surface were made using brightfield and epifluorescence microscopy at different levels of depth, above and below the transwell surface, respectively. Adhesion and transmigration were determined by counting all adherent/transmigrated cells per image and then calculating the number of adherent/transmigrated cells per cellQART transwell (total area, 33.16 mm^2^). Thus, a ratio was expressed by dividing this number by the total number of neutrophils added in each of the eight independent experiments. The following formula was employed for calculation:


$$\frac{total\;neutrophils\;counted}{represented\;area}=\;\frac{x}{33.16\;mm^{2}}.$$

Analogously, the correlation between age and neutrophil transmigration was assessed within the T1D patient subgroup.

### 2.7 Statistical analysis

Neutrophil counts and ROS production levels between the T1D patient groups (wild-type and C1858T *PTPN22* variant) and the control group, under different conditions, e.g., unstimulated, TNF-α, and TNF-α+fMLP, were assessed for normality using the Kolmogorov–Smirnov (KS) test. For data satisfying normality criteria (KS test: p > 0.10), statistical significance was evaluated using one-way ANOVA, followed by Tukey’s multiple comparison test. The analysis of multiple biological replicates was performed using the GraphPad Prism software (version 5, San Diego, CA). Additionally, differences in neutrophil counts and ROS production between the patient and control groups were analyzed using an unpaired t-test. Pearson’s correlation analysis was applied to assess potential age-dependent effects on neutrophil function and transmigration. Values are expressed as means ± SEM. A p-value of less than 0.05 was considered statistically significant. Neutrophil adhesion and transmigration through HUVECs were evaluated using proportion Z-tests. The total sum of neutrophils added, adhered, and transmigrated in each of eight independent experiments was used to compare the different experimental conditions, e.g., with and without TNF-α, by means of the one- or two-proportion Z-test. A result with p < 0.01 was considered statistically significant.

## 3 Results

We tested the hypothesis that the presence of C1858T *PTPN22* variant may affect neutrophil function in a cohort of 27 T1D patients recruited and genotyped for this SNP (**Table 1**). Fifteen patients were heterozygous for variant C1858T, and 12 patients were wild-type. Patients were closely matched for age and sex. There were no significant differences in patient demographics, including disease duration from onset and treatment.

### 3.1 Neutrophil counts in T1D patients

**Figure 1:**
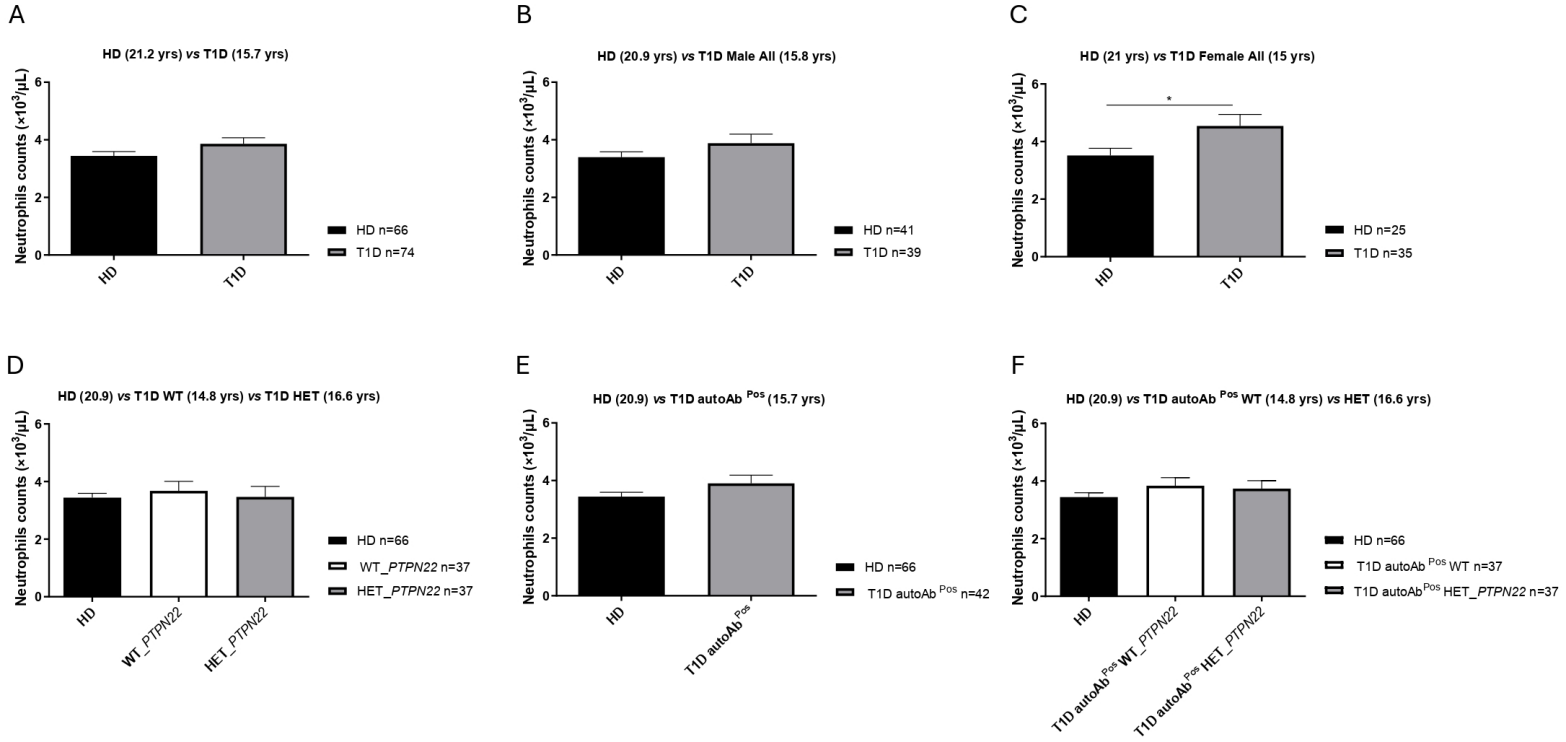
Neutrophil counts in the peripheral blood. Neutrophil counts in type 1 diabetes (T1D) patients (n = 74) *versus* healthy controls (HD) (n = 66) **(A)**, male T1D patients (n = 39) *vs*. male HD (n = 41) **(B)**, female T1D patients (n = 35) *vs*. female HD (n = 25) **(C)**, heterozygous (HET) *PTPN22* T1D patients (n = 37) compared to *PTPN22* wild-type (WT) patients (n = 37) and HD (n = 66) **(D)**, autoantibody-positive T1D patients (n = 42) *vs*. HD (n = 66) **(E)**, and autoantibody-positive HET (C1858T) (n = 37) and WT (C1858C) T1D patients (n = 37) compared to HD (n = 66) **(F)**. Data are expressed as mean ± SEM, with the sample size (n) indicated in the histogram of each respective panel, and mean age (years) is shown in brackets. *p < 0.05.

No significant differences in neutrophil counts were observed when comparing total T1D patients to healthy donor controls or male T1D patients to male healthy donors (**Figures 1A, B**). However, neutrophil counts were significantly higher in female T1D patients compared to female healthy donors (**Figure 1C**). Neutrophil counts did not differ between the T1D subgroups (wild-type or heterozygous for the *PTPN22* variant) and healthy donor controls (**Figure 1D**), between the autoantibody-positive T1D patients and healthy donor controls (**Figure 1E**), or between the heterozygous (C1858T) and wild-type (C1858C) T1D patients and healthy donors (**Figure 1F**).

### 3.2 Enhanced ROS production in neutrophils from C1858T T1D patients

**Figure 2:**
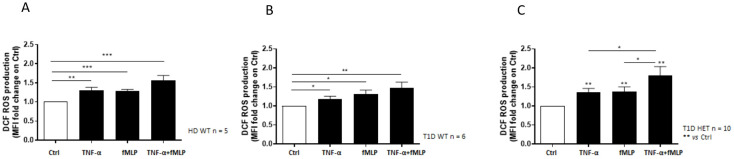
Neutrophil reactive oxygen species (ROS) production measured by DCF fluorescence. Effect of the different stimulatory conditions on ROS production, expressed as mean fluorescence intensity (MFI) fold change in neutrophils isolated from healthy blood donors wild-type (WT) (n = 5) **(A)**, WT type 1 diabetes (T1D) patients (n = 6) **(B)**, and heterozygous (HET) T1D patients (n = 10) **(C)**. Data are expressed as mean ± SEM. *p<0.05, **p<0.01 and ***p<0.001.

**Figure 3:**
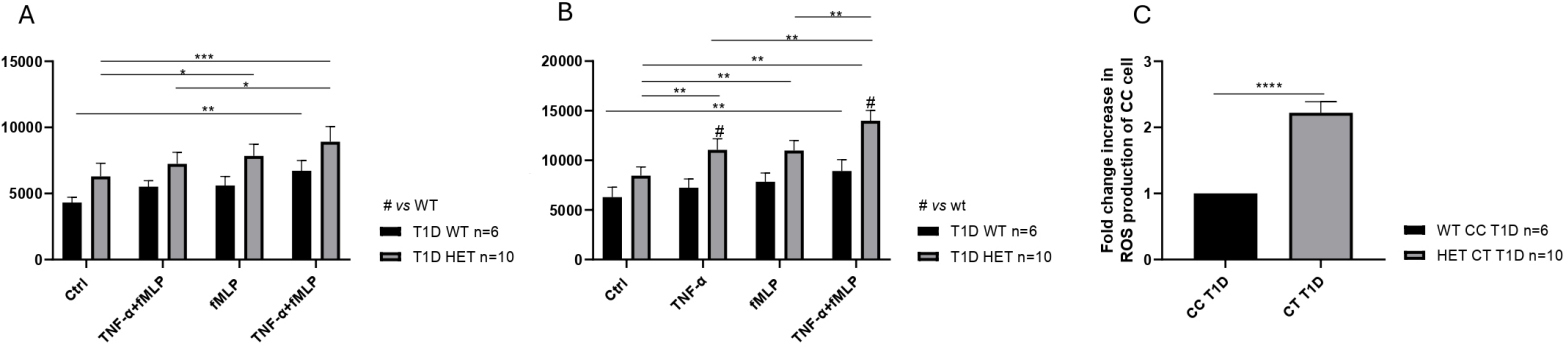
Increased reactive oxygen species (ROS) production in neutrophils of diabetic patients. ROS generation in neutrophils isolated from wild-type (WT) type 1 diabetes (T1D) (n = 6) and healthy controls **(HD)** (n = 5), compared to the respective controls **(A)**. Enhanced mean fluorescence intensity of ROS production in neutrophils from heterozygous (n = 10) compared to wild-type T1D patients (n = 6) and respective controls in response to various stimulatory conditions **(B)**. Increased fold change in ROS production in T1D patients categorized by genotype [heterozygous (HET) C1858T *PTPN22*, n = 10 *vs*. WT C1858C *PTPN22*, n = 6] **(C)**. Data are expressed as mean ± SEM. *p<0.05, **p<0.01, ***p<0.001 and ****p<0.0001.

Neutrophils isolated from the healthy donors WT (n = 5, mean age 31.10 years), *PTPN22* C1858C T1D patients (n = 6, mean age 11.58 years), and *PTPN22* C1858T T1D patients (n = 10, mean age 13.57 years) exhibited a significantly greater ability to produce ROS in response to TNF-α priming, fMLP stimulation, or the combination of TNFα+fMLP (**Figures 2A–C**). Specifically, the combination treatment resulted in significantly higher ROS levels than either TNF-α or fMLP alone. Moreover, these findings were further confirmed by flow cytometric histograms of DCF fluorescence, which showed higher ROS levels in neutrophils from the T1D patient groups (wild-type and C1858T *PTPN22* variant) (n = 16) compared to healthy donors (n = 5) (**Supplementary Figure 2**). As shown in **Figure 3A**, ROS production was increased following combined treatment (TNF-α+fMLP) in both the *PTPN22* C1858C T1D patients (n = 6) and healthy individuals (n = 5) compared to the respective controls (unstimulated). Furthermore, enhanced ROS production in response to priming with TNF-α and then fMLP-treated neutrophils from the *PTPN22* C1858C T1D patients than in those treated with TNF-α or fMLP alone was observed. When the T1D patients were categorized and compared based on their *PTPN22* genotype (WT and HET), differences in ROS production were more evident. ROS levels were significantly higher in the heterozygous T1D patients (n = 10) compared to wild-type T1D patients (n = 6) following treatments with TNF-α and TNF-α+fMLP (p < 0.001). Enhanced ROS levels were observed under fMLP treatment, although this difference was not statistically significant (**Figure 3B**). In addition, fold change in ROS production was markedly increased in the heterozygous C1858T *PTPN22* (n = 10) *vs*. wild-type C1858C *PTPN22* (n = 6) T1D patients (p < 0.05) (**Figure 3C**); although a broader comparison among all genotypes (HD, WT, and HET) revealed a similar trend, the differences were not statistically significant (**Supplementary Figure 3**). A significant negative correlation between age and ROS production was observed across all subjects (HD, WT, and HET) under all tested conditions (**Supplementary Table 3**), likely reflecting the age gap between the adult HD and pediatric T1D patients. In contrast, within the pediatric T1D subgroup (WT + HET), a significant positive correlation with age emerged under TNF-α (r = 0.53, p = 0.033), fMLP (r = 0.49, p = 0.052), and TNF-α+fMLP (r = 0.64, p = 0.008) stimulation, with a similar upward trend under all stimulated conditions (**Supplementary Table 4**). Despite this trend, HET patients—who were on average younger than WT patients (11.58 *vs*. 13.57 years)—exhibited significantly higher ROS levels, indicating that enhanced ROS production is likely driven by the *PTPN22* variant rather than age.

### 3.3 Increased neutrophil adhesion and transmigration in C1858T *PTPN22* T1D patients

**Figure 4:**
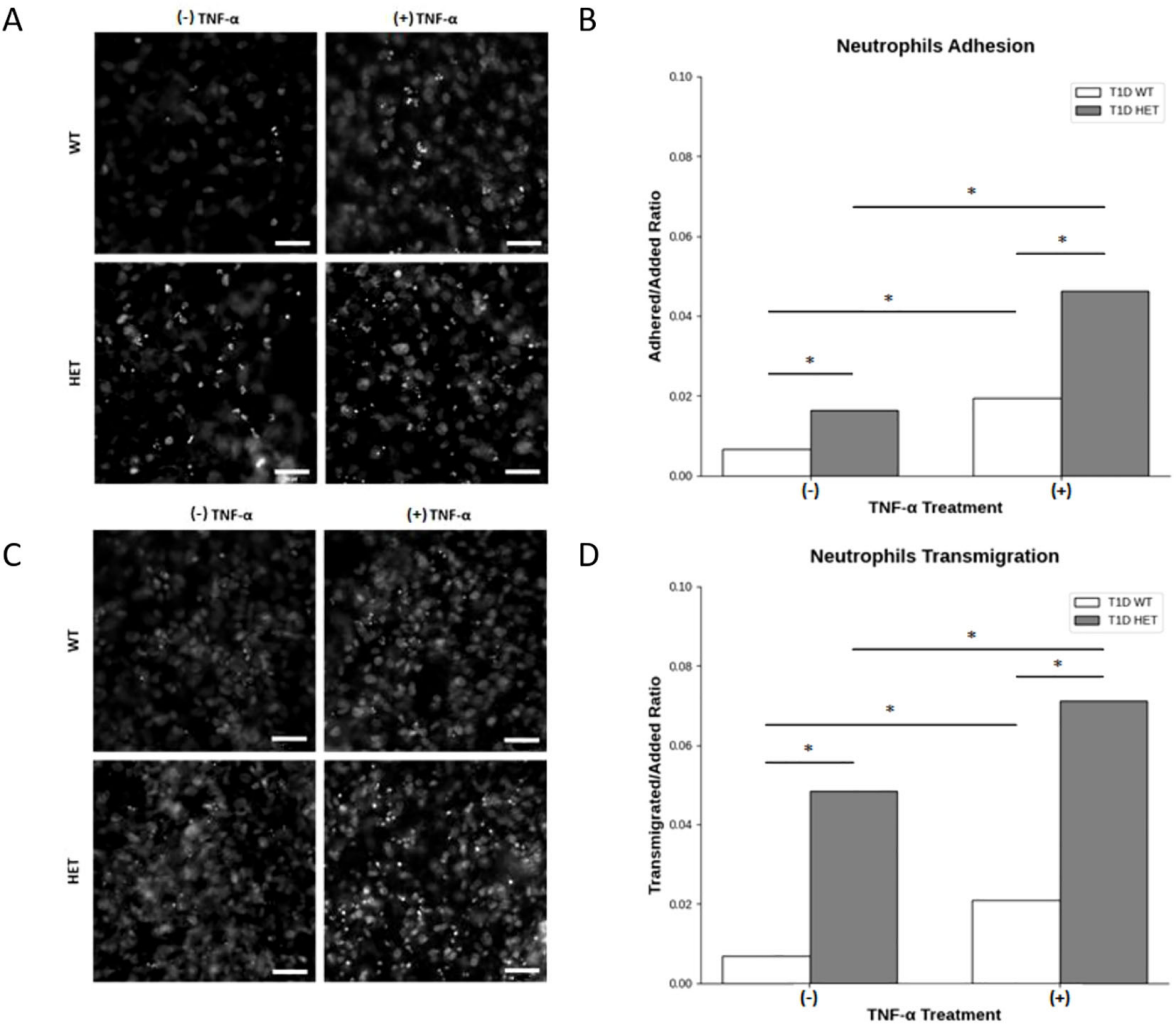
Neutrophil adhesion to human umbilical vein endothelial cells (HUVECs) and transmigration across the endothelial monolayer. Representative phase-contrast microscopy of neutrophils from *PTPN22* wild-type and heterozygous C1858T *PTPN22* type 1 diabetes (T1D) patients adhering to a HUVEC monolayer **(A)** and quantitative comparison of the adherent-to-added neutrophil ratio in heterozygous C1858T *PTPN22* (n = 4) *vs*. wild-type T1D patients (n = 6) in basal and TNF-α-stimulated culture conditions **(B)**. Representative phase-contrast microscopy of neutrophils from *PTPN22* wild-type (WT) and heterozygous (HET) C1858T *PTPN22* T1D patients transmigrating across the HUVEC monolayer **(C)** and comparison of the transmigrated-to-added ratio of neutrophils in heterozygous C1858T *PTPN22* (n = 4) *vs*. wild-type T1D patients (n = 6) in basal and TNF-α-stimulated culture conditions **(D)**. Images and data are representative of n = 8 independent experiments. Bars show the cumulative adhered-to-added and transmigrated-to-added neutrophil ratios across all the experiments. Proportion Z-test, *p < 0.001. Scale bars in A and C, 50 μm.

To investigate whether the presence of the C1858T *PTPN22* variant in T1D patients altered the ability of neutrophils to be recruited to the pancreas, potentially leading to insulitis, the behavior (adhesion and transmigration) of neutrophils isolated from the wild-type (C1858C) and heterozygous (C1858T) T1D patients on endothelial cell monolayers stimulated with TNF-α was examined. Neutrophil adhesion and transmigration were significantly enhanced in the heterozygous C1858T *PTPN22* (n = 4) compared to WT *PTPN22* (n = 6) T1D patients, both under basal conditions and following TNF-α stimulation (**Figure 4**). Specifically, neutrophils from the C1858T patients exhibited higher adhesion (**Figures 4A, B**) and greater transmigration across the monolayer after TNF-α stimulation (**Figures 4C, D**) compared to those from the wild-type T1D patients (Z-test, p < 0.0001). No significant correlation was observed between age and neutrophil transmigration in the pediatric T1D patients carrying (HET) or not carrying (WT) the *PTPN22* C1858T variant (r = −0.21 and −0.05 for unstimulated and TNF-α-stimulated conditions, respectively) (**Supplementary Table 5**). This suggests that the C1858T *PTPN22* variant may enhance neutrophil recruitment and migration independently of age, especially through inflamed endothelium, by contributing to the development of insulitis in T1D.

## 4 Discussion

The main finding from our study demonstrates that the C1858T *PTPN22* variant contributes to enhanced neutrophil activation in T1D. Specifically, neutrophils from the HET C1858T *PTPN22* variant patients exhibited significantly increased ROS production, as well as greater adhesion and transmigration across endothelial cells, compared to those from the WT C1858T *PTPN22* patients. These observations suggest that the C1858T *PTPN22* variant may play a role in amplifying immune cell recruitment during the inflammatory response, independently of age, underscoring the synergistic impact of genetic predisposition and immune dysregulation on neutrophil function in T1D.

Specifically, ROS production tends to increase with age, in contrast to the overall cohort analysis, where a negative correlation was observed, likely reflecting the inclusion of older healthy donors. Notably, this positive age-related trend in T1D was consistent across all stimulated conditions. This pattern reinforces that the observed differences in ROS production between the WT and HET T1D patients are not attributable to age. Crucially, the comparison underlying our primary conclusions involves two pediatric groups with similar mean ages, thereby minimizing age as a confounding factor. Despite being younger on average, HET patients exhibited significantly higher ROS levels, strongly suggesting that the enhanced neutrophil function is likely attributable to the *PTPN22* variant rather than age. Thus, the direction and magnitude of the age effect further support a genotype-driven functional difference rather than an age-related artifact.

Furthermore, adhesion and transmigration of neutrophils through HUVEC monolayers were increased in the heterozygous C1858T *PTPN22 vs*. wild-type *PTPN22* T1D patients, both under basal conditions and following TNF-α stimulation, with no significant correlation with age. These findings suggest that the presence of the C1858T *PTPN22* variant in T1D patients alters the ability of neutrophils to be recruited to the target organ, the pancreas, migrating more rapidly, especially through inflamed endothelium, thus leading to insulitis (32).

Of note, contrasting results were reported on the neutrophil counts in T1D patients at disease onset, found either decreased (17, 33, 34) or increased (35, 36). In the present investigation, neutrophil percentages were similar in the T1D patients at onset and healthy controls. However, neutrophil counts were significantly higher in the female T1D patients than in female healthy controls. Other studies have reported that the size effect was dependent on both age and sex, with diabetic men exhibiting lower circulating neutrophil levels (37). Neutrophil functions were also reported to change at different steps in T1D. Indeed, the current literature is contradictory regarding the host defense functions of neutrophils, including oxidative burst activity (38–40) and migration (41, 42). Increased production of ROS by neutrophils in both T1D patients and rat models (40, 43) not only drives pancreatic β-cell destruction but also compromises neutrophil antioxidant defense, heightening susceptibility to infections in diabetic patients (44). This dysregulated immune response is further compounded by genetic predisposition, such as the C1858 variant of *PTPN22*.

The interplay between oxidative stress and immune dysfunction shows implications in all stages of autoimmune T1D, as described by the literature (45). Specifically, ROS production and impaired neutrophil function not only contribute to cellular injury but also can activate inflammatory, redox-dependent transcription factors, such as NF-κB and cytokine production (46), thereby sustaining inflammation. Therefore, the redox modulation may be more important than once thought, even though newer therapeutic approaches allow for precise targeting of specific immune cells. These results pave the way for future immunotherapeutic strategies targeting the *PTPN22* variant using lipoplexes (47–50). These approaches could effectively mitigate the pathogenetic mechanisms driving diabetic insulitis by modulating not only T- and B-lymphocyte activation but also neutrophil function, including their heightened ROS production, adhesion, and transmigration. These interventions may offer new possibilities to improve outcomes in T1D, considering the multifaceted contribution of neutrophils to disease progression.

## Data Availability

The original contributions presented in the study are included in the article/**Supplementary Material**. Further inquiries can be directed to the corresponding author.
