# Supplementary material for: Effect of the autoimmune-associated genetic variant PTPN22 R620W on neutrophil activation and function in patients with insulin-dependent diabetes mellitus
Source: Front Immunol. 2025 Sep 8;16:1554570. doi: 10.3389/fimmu.2025.1554570 (PMC12450659; doi:10.3389/fimmu.2025.1554570)
Supplement: Supplementary file 1 [file DataSheet1.docx]

Supplementary Material

Effect Of The Autoimmune-associated Genetic Variant *PTPN22* R620W On Neutrophil Activation And Function In Patients With Insulin-dependent Diabetes Mellitus

Eugenia Belcastro^1†^^, Annamaria Cudini^1†^, Mezzani Irene^1^, Stefania Petrini^2^, Valentina D’Oria^2^, Riccardo Schiaffini^1^, Marco Scarsella^1^, Anna Lo Russo^1^, Alessandra Fierabracci^1*^

^1^Bambino Gesù Children’s Hospital, Istituto di Ricovero e Cura a Carattere Scientifico (IRCCS), 00146 Rome, Italy; ^2^Confocal Microscopy Core Facility, Bambino Gesù Children’s Hospital, Istituto di Ricovero e Cura a Carattere Scientifico (IRCCS), 00146 Rome, Italy

† Equally contributed

^ Current address: Department of Translational Research and New Technologies in Medicine and Surgery, University of Pisa, 56126 Pisa, Italy

*** Correspondence:**Corresponding Author
alessandra.fierabracci@opbg.net


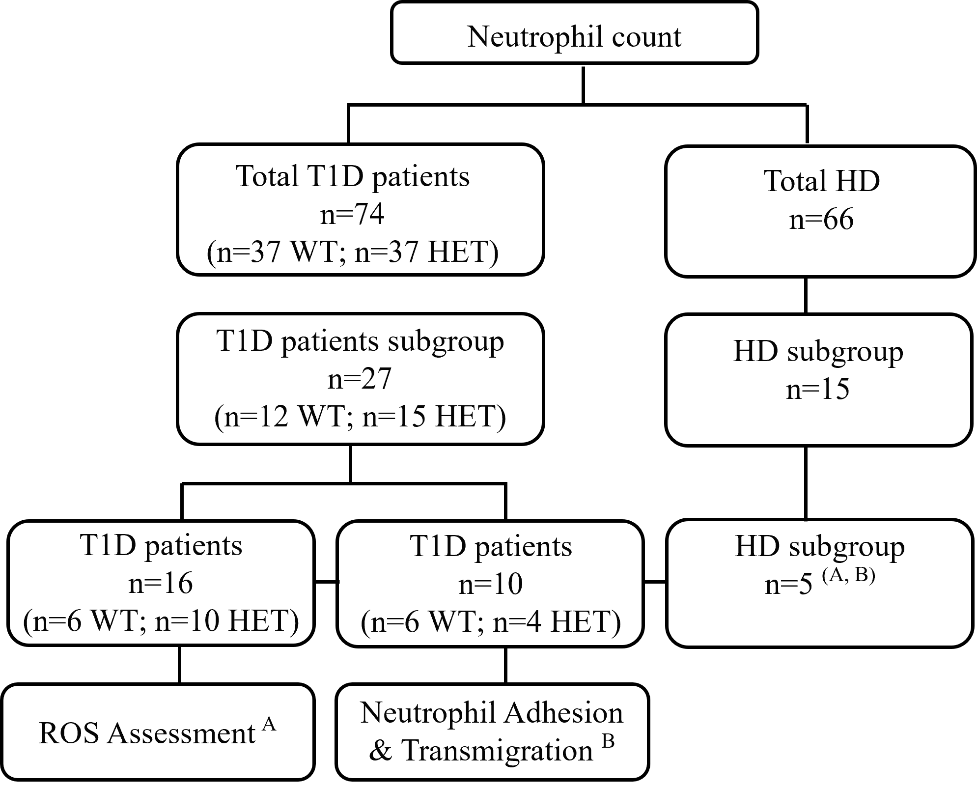


**Supplementary Figure 1. Experimental design.**


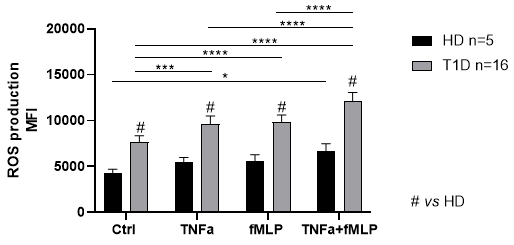


**Supplementary Figure 2. ROS production in T1D patients *versus* healthy donors.** Mean fluorescence intensity for ROS production in T1D *versus* HD under different stimulatory conditions. Data are expressed as mean ± SEM. #p < 0.1, *p<0.05, ***p <0.001, ****p<0.0001.

**Supplementary Figure 3. ROS production levels in HD, T1D WT and T1D HET.** Comparative analysis of ROS production fold change under different stimulatory and control conditions. Data are expressed as mean ± SEM.

**Supplementary Table 1. Clinical characteristics of all T1D patients (n=74) included in the study for neutrophils count..**

| **N°** | **Gender** | **Age (actual years)** | **Age**  **at onset** | **Clinical**  **manifestation** | **Auto Abs** | ***PTPN22***  **genotype** | **C-peptide at onset (ng/ml)** | **HBA1c at onset**  **(mmol/mol)** |
| --- | --- | --- | --- | --- | --- | --- | --- | --- |
| 1 | M | 6.1 | 0.7 | T1D | **GADAb,**  **IAA pos**  IA2Ab borderline  EMA-IgA, TgAb, TPOAb, DGP-IgG Ab,  TRGAb-IgA neg | HET | <0.05 | 62 |
| 2 | F | 18.1 | 7.1 | T1D,  DKA | **IA2Ab pos**  GADAb,  IAA,  EMA-IgA, TgAb, TPOAb, DGP-IgG Ab, TRGAb-IgA neg | HET | 0.53 | 86 |
| 3 | M | 13.8 | 5.7 | T1D | **GADAb, IAA pos**  IA2Ab, TgAb, TPOAb, DGP-IgG Ab, TRGAb-IgA neg | HET | 0.07 | 103 |
| 4 | F | 24.9 | 14.9 | T1D,  Hashimoto's thyroiditis | **GADAb, IAA, IA2Ab pos**  TgAb, TPOAb, DGP-IgG Ab, TRGAb-IgA neg | HET | 0.15 | 110 |
| 5 | F | 17.6 | 7.1 | T1D | **GADAb, IAA pos**  IA2Ab, TgAb, TPOAb, DGP-IgG Ab,  TRGAb-IgA neg | HET | 0.58 | 48 |
| 6 | F | 23.7 | 8.9 | T1D | **GADAbpos**  IAA, IA2Ab, TgAb, TPOAb, DGP-IgG Ab, TRGAb-IgA neg | HET | 0.46 | 80 |
| 7 | M | 12 | 5.2 | T1D | **GADAb,**  **IA2Ab, IAA pos**  TgAb, TPOAb, DGP-IgG Ab, TRGAb-IgA neg | HET | 0.31 | 48 |
| 8 | M | 20.8 | 11.0 | T1D | **GADAb,**  **IAA, IA2Ab pos**  TgAb, TPOAb, DGP-IgG Ab, TRGAb-IgA neg | HET | <0.05 | 87 |
| 9 | F | 16.9 | 7.4 | T1D | **GADAb,**  **IAA pos**  IA2Ab, TgAb, TPOAb, DGP-IgG Ab, TRGAb-IgA neg | HET | 0.15 | 96 |
| 10 | M | 20.4 | 12.6 | T1D | **GADAb,**  **IAA, IA2Ab pos**  TgAb, TPOAb, DGP-IgG Ab, TRGAb-IgG, TRGAb-IgA neg | HET | 0.28 | 54 |
| 11 | M | 11.5 | 4.4 | T1D | **GADAb,**  **IAA, IA2Ab pos**  TgAb, TPOAb, DGP-IgG Ab,  TRG-IgG Ab, TRGAb-IgA neg | HET | 0.54 | 64 |
| 12 | M | 10.6 | 3.9 | T1D | **IAA pos**  GADAb, IA2Ab, TRAb, TgAb, TPOAb, TRGAb-IgA neg | HET | 0.14 | 64 |
| 13 | F | 15.9 | 8.9 | T1D | **GADAb, IAA, IA2Ab pos**  EMA-IgA, ANA, AMA, ASMA, LKM Ab, ARA, APCA, RAb, aCL Ab IgG, aCL Ab IgM, LC1 Ab, TgAb,TPOAb, DGP-IgG Ab, TRGAb-IgA neg | HET | 0.48 | 103 |
| 14 | M | 19.8 | 9.9 | T1D,  severe obesity | **GADAb,**  **IAA, IA2Ab pos**  TgAb, TPOAb, TRGAb-IgA neg | HET | 0.41 | 61 |
| 15 | M | 14.5 | 1.3 | T1D | **GADAb,**  **IAA pos**  IA2Ab, EMA-IgA, TgAb, TPOAb, DGP-IgG Ab,  TRGAb-IgA Ab neg | HET | 0.02 | 65 |
| 16 | M | 19.5 | 8.5 | T1D | **IAA, IA2Ab,**  **TRGAb-IgApos**  GADAb, EMA-IgA, TgAb, TPOAb neg | HET | 0.06 | 75 |
| 17 | F | 19.2 | 7.1 | T1D | **GADAb,**  **IAA, IA2Ab,**  **TPOAbpos**  EMA-IgA, TgAb, DGP-IgG Ab,  TRGAb-IgA neg | HET | 0.31 | 97 |
| 18 | M | 21.1 | 5.9 | T1D, celiac disease | **IA2Ab pos**  GADAb,  IAA, EMA-IgA, TgAb, TPOAb  TRGAb-IgA neg | HET | 0.37 | 88 |
| 19 | M | 19.3 | 7.8 | T1D | **IAA, IA2Ab pos**  GADAb, EMA-IgG, TgAb, TPOAb, DGP-IgG Ab, TRGAb-IgG, TRGAb-IgA neg | HET | 0.42 | 102 |
| 20 | M | 22.7 | 12.5 | T1D | **GADAb, IA2Ab, ANCA pos**  ANA, APCA, anti-dsDNA Ab, aCL Ab IgG, aCL Ab IgM, anti-β2GPI IgG, **anti-β2GPI** IgM, Ab Anti-SS-A/Ro IgG, Ab Anti-SS-B/La IgG, TgAb,  TPOAb, DGP-IgG Ab,  TRGAb-IgA neg | HET | 0.23 | 54 |
| 21 | M | 19.2 | 11 | T1D | **GADAbpos**  IAA, IA2Ab, TgAb, TPOAb, DGP-IgG Ab, TRGAb-IgA neg | HET | 0.12 | 127 |
| 22 | M | 17.8 | 10.1 | T1D,  autoimmune thyroiditis | **GADAbpos**  IAA, IA2Ab, TgAb, TPOAb, TRGAb-IgA neg | HET | 0.75 | 93 |
| 23 | M | 18.3 | 7 | T1D | **GADAbpos**  IA2Ab, TgAb, TPOAb,  TRGAb-IgA neg | HET | 0.15 | 86 |
| 24 | M | 21.9 | 8.8 | T1D,  thyroiditis | **GADAb, IAA, IA2Ab, TgAb, TPOAbpos**  DGP-IgG Ab, TRGAb-IgA neg | HET | 0.45 | 46 |
| 25 | F | 15.9 | 13 | T1D | **GADAb, IAA, ZnT8 Ab pos**  IA2Ab, TgAb, TPOAb, tTG IgA neg | HET | 0.96 | 112 |
| 26 | M | 22.6 | 14.1 | T1D | **GADAb, IAA, IA2Ab pos**  TPOAb, TgAb, DGP-IgG Ab, TRGAb-IgA neg | HET | 0.27 | 109 |
| 27 | F | 21.8 | 14 | T1D,  autoimmune thyroiditis | **GADAb, TPO Ab pos**  IAA, IA2Ab, TgAb, TRGAb-IgA neg | HET | 0.07 | 143 |
| 28 | M | 25.6 | 17.8 | T1D | **GADAb, IAA, IA2Ab pos**  TPOAb, TgAb, DGP-IgG Ab, TRGAb-IgA neg | HET | 0.40 | 94 |
| 29 | F | 20.2 | 13.2 | T1D,  Basedow syndrome | **GADAb, TRAb, TPOAbpos**  IAA, IA2Ab, ADR Ab, TgAb, DGP-IgG Ab,TRGAb-IgA neg | HET | 0.67 | 137 |
| 30 | F | 13.3 | 2.7 | T1D | **GADAb, IAA, IA2Ab, DGP-IgG Ab pos**  TPOAb, TgAb, TRGAb-IgA neg | HET | 0.48 | 73 |
| 31 | F | 9.4 | 4.7 | T1D | **GADAb, IA2Ab pos**  IAA,  TPOAb, TgAb, TRGAb-IgA neg | HET | 0.36 | 133 |
| 32 | F | 12.7 | 10 | T1D | **GADAb, IA2Ab, ZnT8 Ab, TPOAbpos**  IAA, TgAb, TRGAb-IgA neg | HET | 0.79 | 88 |
| 33 | F | 11.5 | 5.77 | T1D | **GADAb, IAA, IA2Ab pos**  TPOAb, TgAb, TRGAb-IgA neg | HET | 0.16 | 74 |
| 34 | F | 21.4 | 8 | T1D | **IAA, IA2Ab, ANA pos**  GADAb, AMA, ASMA, APCA, LKMAb, ARA, RAb, anti-dsDNA, LC1Ab, TPOAb, TgAb, DGP-IgG Ab, TRGAb-IgA neg | HET | 0.08 | 127 |
| 35 | M | 10.1 | 8.2 | T1D | **IA2Ab pos**  GADAb, ZnT8 Ab, IAA, TPOAb, EMA-IgA, TgAb, TRGAb-IgA neg | HET | 0.35 | 101 |
| 36 | M | 20.4 | 7.8 | T1D | **IAA, IA2Ab pos**  GADAb, TPOAb, TgAb, DGP-IgGAb, TRGAb-IgA neg | HET | 0.31 | 143 |
| 37 | M | 16.3 | 1.2 | T1D | **GADAb, IA2Ab pos**  EMA-IgA, TPOAb, TgAb, DGP-IgG Ab,  TRGAb-IgA neg | HET | 0.28 | 90 |
| 38 | F | 18.2 | 8.3 | T1D | **GADAb, IAA, IA2Ab, TgAbpos**  TPOAb, TRGAb-IgA neg | WT | 0.09 | 68 |
| 39 | F | 21.3 | 16.5 | T1D,  nodular Hashimoto’s thyroiditis | **GADAb, TPOAb, TgAbpos**  IAA, IA2Ab, EMA-IgA, TRGAb-IgG,TRGAb-IgA neg | WT | 0.64 | 41 |
| 40 | F | 11.7 | 5.8 | T1D | **GADAb, IA2Ab pos**  TRGAb-IgA weakly pos  IAA, TPOAb, TgAb, DGP-IgG Ab neg | WT | 0.14 | 107 |
| 41 | M | 13.9 | 11.7 | T1D | **GADAb, IA2Ab, ZnT8 Ab pos**  IAA, EMA-IgA, TPOAg, TgAb,  TRGAb-IgA neg | WT | 0.53 | 110 |
| 42 | F | 19.2 | 7.8 | T1D,  obesity | **GADAb, IAA IA2Ab pos**  TgAb, TPOAb,  TRGAb-IgA neg | WT | 0.38 | 102 |
| 43 | M | 19.6 | 8.9 | T1D | **GADAb, IA2Ab, TgAb, TPOAbpos**  TRGAb-IgA weakly pos  IAA, DGP-IgG Ab neg | WT | 0.46 | 100 |
| 44 | M | 17.4 | 12.4 | T1D,  isolated hyperthyrotropine-mia | **IA2Ab pos**  **GADAb, IAA, ZnT8 Ab,** TgAb, TPOAb, TRGAb-IgA neg | WT | 0.97 | 120 |
| 45 | M | 11.3 | 1.2 | T1D,  celiac disease | **GADAb, IAA, IA2Ab, EMA-IgApos**  TPOAb, TgAb, DGP-IgG Ab, TRGAb-IgA neg | WT | 0.09 | 104 |
| 46 | M | 13.7 | 8.2 | T1D | **ANA pos**  GADAb, IAA, IA2Ab, ZnT8 Ab, APCA,  TPOAb,  TgAb, DGP-IgG Ab,  TRGAb-IgA neg | WT | 0.08 | 109 |
| 47 | F | 16 | 4.2 | T1D | **GADAb, IAA, IA2Ab pos**  TPOAb, TgAb, DGP-IgG Ab, DGP-IgG Ab, TRGAb-IgA neg | WT | 0.42 | 98 |
| 48 | F | 22 | 12.3 | T1D | **GADAb, IAA, IA2Ab pos**  TPOAb, TgAb,  TRGAb-IgA neg | WT | 0.28 | 113 |
| 49 | F | 9 | 5.6 | T1D | **IA2Ab pos**  GADAb, IAA, TPOAb, TgAb, DGP-IgG Ab, TRGAb-IgA neg | WT | 0.34 | 118 |
| 50 | F | 11.5 | 6.6 | T1D | **GADAb, IAA, IA2Ab pos**  TPOAb, TgAb,  TRGAb-IgA neg | WT | 0.44 | 99 |
| 51 | M | 17.1 | 6 | T1D | **IAA, IA2Ab pos**  GADAb, TPOAb,  TgAb,  TRGAb-IgA neg | WT | 0.84 | 54 |
| 52 | F | 20.6 | 11.5 | T1D,  obesity | **GADAb, IAA pos**  IA2Ab,  TPOAb, TgAb,  DGP-IgG Ab, TRGAb-IgA neg | WT | 0.35 | 106 |
| 53 | M | 12.7 | 2.5 | T1D | **GADAb, IAA, IA2Ab pos**  TPOAb, TgAb, DGP-IgG Ab,  TRGAb-IgA neg | WT | 0.24 | 63 |
| 54 | F | 14.8 | 5.2 | T1D | **IAA, IA2Ab, TPOAbpos**  GADAb, TgAb,  TRGAb-IgA neg | WT | 0.28 | 96 |
| 55 | F | 12 | 9.5 | T1D | **GADAbpos**  IAA, IA2Ab, ZnT8 Ab, TPOAb, EMA-IgG, DGP-IgG Ab, TRGAb-IgG, TRGAb-IgA neg | WT | 2.16 | 58 |
| 56 | F | 12.3 | 5.7 | T1D | **GADAb, TPOAb, TgAbpos**  IAA, EMA-IgA,  TRGAb-IgA, neg | WT | 0.11 | 98 |
| 57 | F | 19.2 | 10.4 | T1D | **IAA pos**  GADAb, IA2Ab,  TPOAb, TgAb, EMA-IgA, DGP-IgG Ab, TRGAb-IgA neg | WT | 0.18 | 146 |
| 58 | M | 10.2 | 2.7 | T1D | **GADAbpos**  IAA,  TPOAb, TgAb, EMA-IgA, DGP-IgG Ab,  TRGAb-IgA neg | WT | <0.05 | 87 |
| 59 | F | 16.2 | 2.4 | T1D, hypertriglyceride-mia,  MTHFR heterozygosity, celiac disease, microcytic anemia | **GADAb, IA2Ab pos**  IAA, TPOAb, TgAb, EMA-IgA, DGP-IgG Ab, TRGAb-IgA neg | WT | 0.17 | 72 |
| 60 | M | 17.3 | 11,9 | T1D | **GADAb, IAA pos**  TPOAb, TgAb,  DGP-IgG Ab, TRGAb-IgG, TRGAb-IgA neg | WT | 0.37 | 107 |
| 61 | M | 8.9 | 2.9 | T1D | **IAA, IA2Ab pos**  GADAb, TPOAb, TgAb, EMA-IgA, DGP-IgG Ab,  TRGAb-IgA neg | WT | <0.05 | 60 |
| 62 | F | 9.4 | 5.8 | T1D | **GADAb, IA2Ab pos**  **TRGAb-IgA weakly pos**  IAA, TPOAb, DGP-IgG Ab neg | WT | 0.19 | 93 |
| 63 | M | 13.2 | 8.3 | T1D | **IAA, DGP-IgG Ab pos**  **TRGAb-IgA weakly pos**  GADAb, IA2Ab, TPOAb, TgAb, EMA-IgA, TRGAb-IgG neg | WT | 0.45 | 76 |
| 64 | M | 19.4 | 13.6 | T1D | **GADAb, IAA pos**  TPOAb, TgAb,  TRGAb-IgA neg | WT | 0.34 | 110 |
| 65 | M | 19 | 10.2 | T1D | **GADAb, IAA, IA2Ab pos**  ANA, TPOAb, TgAb, EMA-IgA,  TRGAb-IgA neg | WT | 0.24 | 97 |
| 66 | M | 10.3 | 8.6 | T1D | **GADAb, IAA, IA2Ab, ZnT8 Ab pos**  TPOAb, EMA-IgA, TRGAb-IgA neg | WT | 0.13 | 59 |
| 67 | M | 6.2 | 0.8 | T1D | **IAA, IA2Ab pos**  GADAb, TPOAb, TgAb, EMA-IgA, DGP-IgG Ab, TRGAb-IgA neg | WT | 0.17 | 71 |
| 68 | M | 14.2 | 8.4 | T1D | IAA pos  GADAb, IA2Ab borderline  TPOAb, EMA-IgA,  TRGAb-IgA neg | WT | 0.78 | 79 |
| 69 | M | 10.6 | 7.4 | T1D,  DKA | **IA2Ab pos**  GADAb, IAA, ZnT8 Ab, TPOAb, TgAb, EMA-IgA, TRGAb-IgA neg | WT | NA | 55 |
| 70 | F | 22.2 | 15.5 | T1D | **GADAb, IAA, IA2Ab pos**  TPOAb, TgAb, EMA-IgA, DGP-IgG Ab, TRGAb-IgA neg | WT | 0.19 | 120 |
| 71 | F | 20 | 9.8 | T1D | **GADAb, IAA pos**  IA2Ab, TPOAb, TgAb, EMA-IgA, DGP-IgG Ab, TRGAb-IgA neg | WT | 0.17 | 143 |
| 72 | F | 13.7 | 3.1 | T1D | **GADA, IA2Ab, CAb, β2GP-1Ab, anti-phospholipid pos**  IAA,TgAb, TPOAb, TRGAb, DGP-IgGAb, EMA-IgA, LKMAb, LC1Ab, ANA, anti-DNA, ENA, RAb, ARA, AMA, ASMA, APCA, ASMA, APCA, anti-adrenal neg | WT | 0.27 | NA |
| 73 | F | 15 | 8.7 | T1D | **GADA, IA2Ab, IAA pos**  TgAb, TPOAb, TRGAb, TPOAb, TRGAb, EMA neg | WT | 0.51 | 84 |
| 74 | M | 15 | 10.4 | T1D | **GADA, IA2Ab pos**  IAA, ZnT8, TgAb, TPOAb, TRGAb, DGP-IgGAb neg | WT | 0.25 | 128 |

NA= not available; M = Male; F = Female

**Supplementary Table 2. List of all HD subjects (n=66) included in the study.**

| **N°** | **Gender** | **Age** |  | **N°** | **Gender** | **Age** |
| --- | --- | --- | --- | --- | --- | --- |
| 1 | M | 18 |  | 34 | M | 19 |
| 2 | M | 23 |  | 35 | M | 23 |
| 3 | M | 19 |  | 36 | M | 24 |
| 4 | M | 23 |  | 37 | M | 21 |
| 5 | M | 23 |  | 38 | M | 20 |
| 6 | M | 21 |  | 39 | M | 23 |
| 7 | M | 20 |  | 40 | M | 21 |
| 8 | M | 18 |  | 41 | M | 21 |
| 9 | M | 22 |  | 42 | F | 22 |
| 10 | M | 18 |  | 43 | F | 22 |
| 11 | M | 23 |  | 44 | F | 21 |
| 12 | M | 19 |  | 45 | F | 22 |
| 13 | M | 22 |  | 46 | F | 22 |
| 14 | M | 22 |  | 47 | F | 23 |
| 15 | M | 18 |  | 48 | F | 23 |
| 16 | M | 22 |  | 49 | F | 21 |
| 17 | M | 19 |  | 50 | F | 21 |
| 18 | M | 22 |  | 51 | F | 21 |
| 19 | M | 22 |  | 52 | F | 19 |
| 20 | M | 19 |  | 53 | F | 21 |
| 21 | M | 20 |  | 54 | F | 19 |
| 22 | M | 22 |  | 55 | F | 21 |
| 23 | M | 20 |  | 56 | F | 20 |
| 24 | M | 23 |  | 57 | F | 20 |
| 25 | M | 23 |  | 58 | F | 20 |
| 26 | M | 22 |  | 59 | F | 23 |
| 27 | M | 24 |  | 60 | F | 22 |
| 28 | M | 19 |  | 61 | F | 18 |
| 29 | M | 23 |  | 62 | F | 21 |
| 30 | M | 20 |  | 63 | F | 19 |
| 31 | M | 20 |  | 64 | F | 18 |
| 32 | M | 20 |  | 65 | F | 22 |
| 33 | M | 19 |  | 66 | F | 22 |

M = Male; F = Female

**Supplementary Table 3. Pearson correlation (r) between age and ROS levels across all subjects (HD, WT, HET).**

| **Condition** | | **Pearson_r** | **p_value** | |  |
| --- | --- | --- | --- | --- | --- |
| ROS_Unstim | -0.47294286653480294 | | | 0.030369113039914103 | |
| ROS_TNF-α | -0.44643091594740086 | | | 0.04249092660758957 | |
| ROS_fMLP | -0.49515178771886853 | | | 0.022474462040408077 | |
| ROS_TNF- α +fMLP | -0.49594244594766584 | | | 0.022227078444873077 | |

**Supplementary Table 4. Pearson correlation (r) between age and ROS levels** **within the paediatric T1D subgroup (WT + HET).**

| **Condition** | **Pearson_r** | **p_value** | |
| --- | --- | --- | --- |
| ROS_Unstim | 0.3893694823488826 | | 0.13603747182599302 |
| ROS_TNF-α | 0.5343715557565195 | | 0.032974184454140874 |
| ROS_fMLP | 0.49445844517957627 | | 0.0515373287426861 |
| ROS_TNF-a+fMLP | 0.6400193732609492 | | 0.007577020765334663 |

**Supplementary Table 5.**  **Pearson correlation (r) between age and neutrophil transmigration in paediatric T1D subgroup (WT + HET).**

| **Condition** | **Pearson_r** | **p_value** |
| --- | --- | --- |
| Neutrophil Migration (-) TNF-α | -0.20659278192983924 | 0.5668776476374265 |
| Neutrophil Migration (+) TNF-α | -0.051058216295065156 | 0.8886008655958901 |
